# Novel insertion in exon 5 of the *TCOF1* gene in twin sisters with Treacher Collins syndrome

**DOI:** 10.1007/s13353-012-0091-3

**Published:** 2012-03-14

**Authors:** Bożena Anna Marszałek-Kruk, Piotr Wójcicki, Robert Śmigiel, Wiesław H. Trzeciak

**Affiliations:** 1 Department of Genetics, Wrocław University of Environmental and Life Sciences, ul. Kożuchowska 7, 51-631 Wrocław, Poland; 2 Department of Plastic Surgery, Wrocław Medical University, Wrocław, Poland; 3 Department of Genetics, Wrocław Medical University, Wrocław, Poland; 4 Faculty of Public Health WSPiA, Poznan, Poland

**Keywords:** *TCOF1*, Novel insertion, Premature termination, Treacher Collins syndrome

## Abstract

Treacher Collins syndrome (TCS) is associated with an abnormal differentiation of the first and second pharyngeal arches during fetal development. This causes mostly craniofacial deformities, which require numerous corrective surgeries. TCS is an autosomal dominant disorder and it occurs in the general population at a frequency of 1 in 50,000 live births. The syndrome is caused by mutations in the *TCOF1* gene, which encodes the serine/alanine-rich protein named Treacle. Over 120 mutations of the *TCOF1* gene responsible for TCS have been described. About 70% of recognized mutations are deletions, which lead to a frame shift, formation of a termination codon, and shortening of the protein product of the gene. Herewith, a new heterozygotic insertion, c.484_668ins185bp, was described in two monozygotic twin sisters suffering from TCS. This mutation was absent in their father, brother, and uncle, indicating a de novo origin. The insertion causes a shift in the reading frame and premature termination of translation at 167 aa. The novel insertion is the longest ever found in the *TCOF1* gene and the only one found among monozygotic twin sisters.

Treacher Collins syndrome (TCS) [Online Mendelian Inheritance in Man (OMIM) 154500; http://www.omim.org/entry/154500], also referred to as Franceschetti syndrome, is an autosomal dominant disorder affecting differentiation of the first and second pharyngeal arches, and is classified as a mandibulofacial dysostosis (MFD1) (Metro 1965). It occurs in the general population at a frequency of 1 in 50,000 live births. In 60% of cases, the syndrome is caused by de novo mutations, while in the other 40%, it has a family history (Jones et al. 1975).

Abnormal differentiation of the pharyngeal arches in TCS is caused mainly by mutations in the *TCOF1* gene (Treacher Collins-Franceschetti 1). The gene is comprised of 25 exons, 49 to 561 bp in length. In 2004, So et al. (2004) discovered two additional exons: 6A of length 231 bp, situated between exons 6 and 7, and 16A, 108 bp in length, localized between exons 16 and 17.

The *TCOF1* gene transcript encodes a protein named Treacle, consisting of 1411aa (Valdez et al. 2004), while the transcript containing the additional exon 6A encodes a protein which is longer by 77aa (So et al. 2004). Treacle participates in the formation of pre-rRNA by interacting with UBF (upstream binding factor) and binding to the UCE (upstream control element) sequence, as well as to the main promoter region, causing DNA looping during the transcription process (Valdez et al. 2004).

However, in some patients with TCS, no mutations in the *TCOF1* gene were found. In these individuals, mutations in the *POLR1C* and *POLR1D* genes encoding a subunit of RNA polymerases I and III have recently been detected (Dauwerse et al. 2011), suggesting that TCS is genetically heterogeneous and results from a defect in ribosome biogenesis.

TCS is attributed to haploinsufficiency or a negative dominant effect of the mutated protein. Over 120 mutations of the *TCOF1* gene, responsible for TCS, have been described so far (Gladwin et al. 1996; Wise et al. 1997; Splendore et al. 2000; Ellis et al. 2002; Marszałek et al. 2003; Dixon et al. 2004; Su et al. 2006). It has been shown that the reported diversity of patients’ phenotypes does not depend on the type and localization of the mutation (Splendore et al. 2000).

This report describes clinical findings, molecular diagnostics, and treatment of monozygotic twin sisters with typical symptoms of TCS, operated upon at the Department of Plastic Surgery of Wrocław Medical University, Poland. The research described was reviewed and approved by the relevant bioethical committee.

Twin sisters, 18 years of age at the time of analysis, were born at full term from young, healthy, unrelated parents who did not show any apparent craniofacial abnormalities. Clinical symptoms were identical in both sisters and included downward slanting of the eyelids (anti-mongolian slant), hypoplasia of the zygomatic bone, mandibular hypoplasia, coloboma with eyelashes absent in the medial part of the eyelids, microtia (underdevelopment of the auricles), and hypoplasia of the middle ear with concomitant atresia of the external auditory canal. On the basis of clinical symptoms, TCS was diagnosed. Both sisters underwent several operations, including reconstruction of the ears, reconstruction of the zygomatic bone and lateral margins of the orbits using spongy iliac bone grafts, eyelid correction by means of Z-plasty and long musculocutaneous flap transpositions from the upper lids, rhinoplasty, and chin osteotomy using Obwegeser’s method (Fig. 1c–f).

**Fig. 1:**
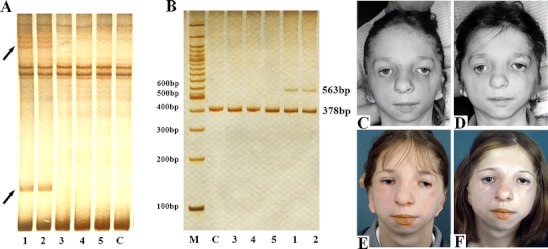
Electrophoretic analysis of DNA and photographs of monozygotic twin sisters with Treacher Collins syndrome (TCS). **a** Multitemperature single-strand conformation polymorphism (MSSCP) analysis of the amplified exon 5 of *TCOF1*. The *arrow* indicates samples which demonstrate changes in the electrophoretic mobility of single-stranded and double-stranded DNA fragments. **b** Electrophoretic separation of the amplified fragment of exon 5 in 8% polyacrylamide gel. Lanes *1* and *2*, patients; *3*, patients’ brother; *4*, patients’ father; *5*, patients’ uncle; *C*, healthy subject; *M*, size marker. **c** Patient 1: preoperative view at 13 years of age. **d** Patient 2: preoperative view at 13 years of age. **e** Patient 1: postoperative view at 16 years of age. **f** Patient 2: postoperative view at 16 years of age

DNA was isolated from peripheral blood leukocytes of the two patients and their healthy brother, father, and uncle. We were unable to obtain genetic material from the patients’ mother because she had died before the time of sampling. Exons 1 through 27 of *TCOF1*, including exon–intron borders, were amplified by polymerase chain reaction (PCR) under optimal conditions, using specific primers. The PCR products were subjected to multitemperature single-strand conformation polymorphism (MSSCP) analysis in 8% polyacrylamide gel at 5°C, 15°C, and 25°C, using the DNA Pointer Mutation Detection System. The electrophoresis was followed by silver staining.

Fragments of the allele which exhibited an abnormal MSSCP pattern were separated from the normal allele by electrophoresis in 8% polyacrylamide gel. The fragments removed from the gel were eluted in 1× TAE buffer at 75°C for 2 h. After elution, the fragments were used as templates for the reamplification of single bands by PCR. The PCR products were purified on the DNA Gel Out columns (A&A Biotechnology, Poland), followed by direct sequencing with the use of a BigDye v3.0 Terminator Cycle Sequencing Kit and specific primers. The dideoxy-terminated fragments were identified by capillary gel electrophoresis based on the ABI 310 DNA Analysis System.

The MSSCP analysis of the amplified fragments of exon 5 of the *TCOF1* gene (Fig. 1a) demonstrated changes in the electrophoretic mobility of the single- and double-stranded DNA in the two patients, while the changes were observed in neither in the patients’ father, brother, or uncle, nor in 154 healthy subjects. Electrophoretic separation in 8% polyacrylamide gel (Fig. 1b) revealed striking differences in the migration of the amplified fragments. In order to confirm the results obtained in the MSSCP analysis and in the separation of 8% polyacrylamide gel, direct sequence analysis was performed of the normal and mutated allele fragments.

The analysis on the inserted fragment demonstrated a novel, heterozygotic insertion: c.484_668ins 5′-TCATCCTTATCTTTATAGAAAAGTAAACTGAGGCCCAGAGAGGTGAAGGGACTAAGATCCCACAACTGGTGACACAGGTTGAGCTCTGAATCCAGGTGGGGCAATTACTCACAACCCTATCCTTCTCTGACATGTAAGACTGTCCCCTACCAGGCACCTTAAATACTACGTTGGTCTCAGAAACT-3′.

Analysis of the novel insertion with the use of the OMIGA 2.0 system allows an assumption that it causes a premature termination of translation at 167aa, which results in the formation of a protein product of the gene devoid of the nuclear localization signal.

A majority of mutations responsible for TCS are localized in exons, mainly in the hot spots in exons 10, 15, 16, 23, and 24 (Splendore et al. 2000). However, relatively few mutations were described in exon 5. The most commonly occurring mutations of the *TCOF1* gene include deletions, from 1 to 40 bp, which cause a shift of the reading frame, formation of the termination codon, and shortening of the protein product devoid of the nuclear localization signal. The next most common mutations of the *TCOF1* gene are insertions, most frequently localized in exons 9, 10, and 23.

In the investigated patients, a novel, heterozygotic insertion, c.484_668ins185bp, was detected in two monozygotic twin sisters. This mutation was absent in the patients’ father, brother, and uncle, which probably indicates a de novo origin, since the examination of the patients’ mother was not possible. The c.484_668ins185bp insertion causes a reading-frame shift and premature termination of translation at 167aa. The novel c.484_668ins is the longest (185-bp) insertion discovered in the *TCOF1* gene.

We believe that these findings facilitated a precise diagnosis of both patients and extended our knowledge of the pathogenesis of TCS.

Molecular diagnostics play a significant role for patients with TCS, in both the prenatal and postnatal stages, and has an undeniable impact on the development of genetic counseling. Molecular evaluation is of extreme importance for families with a history of TCS, including both examined sisters, to determine the risk of the disease in their offspring. Presently, the prenatal diagnostics of TCS is carried out by means of fetoscopy or ultrasound evaluation (Cohen et al. 1995). Although non-invasive ultrasound techniques have progressed recently, the diagnosis of TCS *intra-utero* is very difficult and mild TCS cases may remain undiagnosed. On the other hand, molecular examinations may be performed in the first trimester and provide an accurate diagnosis. This type of diagnostics may be conducted in cases such as the twin sisters examined in this paper.
